# Editorial: Leveraging digital and technological innovations in cardio-oncology: building collaborative networks, implementing education and improving the cardiac outcomes of patients

**DOI:** 10.3389/fcvm.2023.1184988

**Published:** 2023-04-26

**Authors:** Abdulaziz Hamid, James MacLeod, Samuel Erb, Generika Berman, Hugo R. Martinez, Marielle Scherrer-Crosbie, Richard K. Cheng, Sherry-Ann Brown

**Affiliations:** ^1^ Medical College of Wisconsin, Milwaukee, WI, United States; ^2^ Medical College of Wisconsin, Green Bay, WI, United States; ^3^ Department of Pediatric Cardiology, University of Tennessee Health Science Center, Memphis, TN, United States; ^4^ Department of Pediatric Medicine, St. Jude Children's Research Hospital, Memphis, TN, United States; ^5^ Division of Cardiovascular Diseases, Department of Medicine, Hospital of the University of Pennsylvania, Philadelphia, PA, United States; ^6^ Cardio-Oncology Program, Division of Cardiology, University of Washington, Seattle, WA, United States; ^7^ Cardio-Oncology Program, Division of Cardiovascular Medicine, Medical College of Wisconsin, Milwaukee, WI, United States; ^8^ Department of Cardiovascular Diseases, Mayo Clinic, Rochester, MN, United States

**Keywords:** artificial intelligence, telemedicine, precision medicine, digital health, health equity, cardio-oncology

**Editorial on the Research Topic** Leveraging digital and technological innovations in cardio-oncology: Building collaborative networks, implementing education and improving the cardiac outcomes of patients

## Introduction

The aging population and improvements in cancer detection and treatment have contributed to a growing population of cancer survivors. There were approximately 1.9 million newly diagnosed cancer cases in 2022 and more than 600,000 cancer deaths in the United States. Cancer patients are at a higher risk of dying from cardiovascular disease (CVD) than the general population (1–3). Thus, cardio-oncology has developed as a new subspecialty that focuses on the prevention and management of CVD associated with cancer or resulting from cancer treatments (Brown). As we transition into the digital era, we must leverage digital and technological innovations in cardio-oncology to further improve the outcomes of cancer patients. This focused issue explores the potential application of telemedicine, artificial intelligence (AI), precision medicine, and big data in the growing field of cardio-oncology. These manuscripts are highlighted below**.**

Batalik et al. examine cardio-oncology rehabilitation methods and further investigate the usefulness of telemedicine services as a viable option to increase patient access to cardio-oncology rehabilitation. There is a well-established link between chemotherapy and cardiotoxicities in patients who have received cancer treatment (4). The need for cardio-oncology rehabilitation, a model of comprehensive cardiac rehabilitation focused on the unique care of cancer patients and survivors, has been amplified with recent studies displaying more patients with coexisting risk factors that further exacerbate cancer therapy-related cardiotoxicity (5). However, the availability of cardio-oncology rehabilitation has been met by challenges such as the COVID-19 pandemic, which restricted elective healthcare, and long distances to cardio-oncology centers (6, 7).

Kappel et al. explore the possible role that telehealth and AI could play in the delivery of cardio-oncology care to rural and underserved communities. The authors note over the last ten years, there has been a rise in cardio-oncology clinics worldwide, mostly in larger academic and urban centers. Providing cardio-oncology care in rural communities, however, is met with many challenges. Accessibility to health care is one of the main drivers of healthcare disparities faced by rural as compared to urban communities (8). The authors emphasize the importance of a multidisciplinary team of cardiologists, oncologists, and other healthcare professionals in cardio-oncology. Thus, the authors proposed a telehealth model that incorporates AI in cardio-oncology. The proposed telehealth model consists of three phases: pre-treatment, during treatment, and post-treatment, and allows for remote communication and monitoring during each step in the care of the cancer patient.

Chen et al. explain how as cancer survivors are living longer after receiving various cancer therapies, the cardiovascular impact of these interventions is becoming apparent. The authors emphasize the importance of improved risk stratification and disease screening in patients receiving oncological treatment to assess for and to prevent cardiovascular complications. As noted by the authors, there are vast stores of data that can be harnessed to better ascertain the risk for cardiovascular complications, such as surveillance imaging and other diagnostic testing data. The authors discuss advances in AI that allow early detection and identification of cardiac risk through the analysis of cardiac magnetic resonance imaging, computed tomography, echocardiograms, and electrocardiograms. The authors examine studies demonstrating how AI-guided cardiovascular image analysis can aid in the detection of risk and disease features to provide opportunities for expedited intervention to prevent adverse cardiovascular outcomes in patients undergoing cancer therapies. These concepts in AI inspire possibilities for new efficiency, customization, and efficacy with the potential to revolutionize cardio-oncology patient care.

Sadler et al. detail how AI, precision medicine, and machine learning are innovative tools being used to improve CVD-related outcomes in cardio-oncology. This manuscript discusses these resources giving examples of their use and how they interact to create personalized treatment plans on an individual basis, serving as an effective alternative to the one-size-fits-all approach (9). A notable point raised is that existing health technologies may not have sufficient representative use from minorities to obtain accurate results for these groups. In recognizing this, precision cardio-oncology can bridge gaps in care by continuing to gather data on underrepresented groups and updating technologies regularly so a personalized approach can be applied equitably (10).

Brown et al. describe how cardiologists, oncologists, and other specialists and consultants came together during the Cardiology Oncology Innovation Network 2021 ThinkTank to combat cardiovascular toxicity complications of evolving cancer therapies. Participants collaborated to address knowledge gaps, facilitate the development and implementation of innovations and cross-platform communication, and expand the field of cardio-oncology in addressing digital transformation and health equity. Focus was placed on furthering prevention efforts, tackling health inequity, and strengthening interdisciplinary collaboration between cardiologists and oncologists in cardio-oncology. Experts discussed cardio-oncology topics, including digital health and AI, informatics in the global cardio-oncology registry, innovation in education as well as future aims. This model of information exchange and the ideas described inspire collaboration within the fields involved as well as further exploration into all spheres touched by patient care, education, and technology.

Industry and academia are becoming intertwined more than ever. In this case-based manuscript, MacLeod et al. examine the elements of creating an academic-industry partnership in digital health that is applicable to all medical specialties. Ten steps that were taken to establish a partnership with a digital health start-up are discussed, thus providing real-world examples of the described methodology. It could be helpful to academics preparing to do digital health research, digital health businesses interested in academic partnerships, clinicians implementing digital health, users of these technologies, and other partners. This manuscript aims to ensure fairness in digital health research, advance partnerships and collaborations, and educate students at all levels of training. This manuscript builds on others that outline methods to build academic-industry collaboration and is meant to help kickstart further manuscripts outlining this process and the research products that come from similar collaborations.

## Conclusion

Through exploring the roles and optimal implementation of telehealth, AI, precision medicine, and academic-industry collaboration, this focused issue covers many of the actionable and pertinent topics in cardio-oncology research today. The organizing authors, physicians, and researchers strive to promote the adoption of innovations by cancer patients, survivors, institutions, and clinics. We aim to significantly enhance the cardiovascular health of cancer patients and survivors at risk of developing CVD through our common goals of prevention and innovation.
